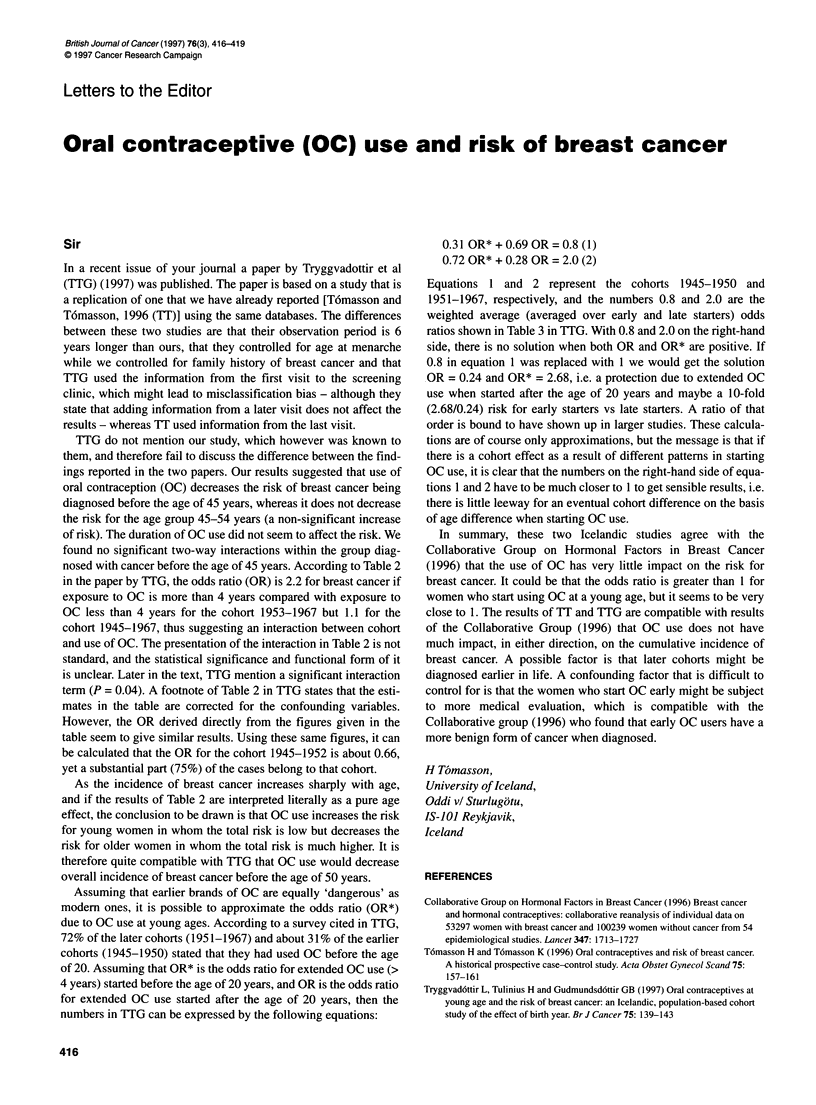# Oral contraceptive (OC) use and risk of breast cancer.

**DOI:** 10.1038/bjc.1997.400

**Published:** 1997

**Authors:** H. TÃ¶masson


British Journal of Cancer (1997) 76(3), 416–419
© 1997 Cancer Research Campaign

Letters to the Editor

### Oral contraceptive (OC) use and risk of breast cancer

**Sir**

In a recent issue of your journal a paper by Tryggvadottir et al (TTG) (1997) was published. The paper is based on a study that is a replication of one that we have already reported [Tómasson and Tómasson, 1996 (TT)] using the same databases. The differences between these two studies are that their observation period is 6 years longer than ours, that they controlled for age at menarche while we controlled for family history of breast cancer and that TTG used the information from the first visit to the screening clinic, which might lead to misclassification bias – although they state that adding information from a later visit does not affect the results – whereas TT used information from the last visit.

TTG do not mention our study, which however was known to them, and therefore fail to discuss the difference between the findings reported in the two papers. Our results suggested that use of oral contraception (OC) decreases the risk of breast cancer being diagnosed before the age of 45 years, whereas it does not decrease the risk for the age group 45–54 years (a non-significant increase of risk). The duration of OC use did not seem to affect the risk. We found no significant two-way interactions within the group diagnosed with cancer before the age of 45 years. According to Table 2 in the paper by TTG, the odds ratio (OR) is 2.2 for breast cancer if exposure to OC is more than 4 years compared with exposure to OC less than 4 years for the cohort 1953–1967 but 1.1 for the cohort 1945–1967, thus suggesting an interaction between cohort and use of OC. The presentation of the interaction in Table 2 is not standard, and the statistical significance and functional form of it is unclear. Later in the text, TTG mention a significant interaction term ($P = 0.04$). A footnote of Table 2 in TTG states that the estimates in the table are corrected for the confounding variables. However, the OR derived directly from the figures given in the table seem to give similar results. Using these same figures, it can be calculated that the OR for the cohort 1945–1952 is about 0.66, yet a substantial part (75%) of the cases belong to that cohort.

As the incidence of breast cancer increases sharply with age, and if the results of Table 2 are interpreted literally as a pure age effect, the conclusion to be drawn is that OC use increases the risk for young women in whom the total risk is low but decreases the risk for older women in whom the total risk is much higher. It is therefore quite compatible with TTG that OC use would decrease overall incidence of breast cancer before the age of 50 years.

Assuming that earlier brands of OC are equally 'dangerous' as modern ones, it is possible to approximate the odds ratio (OR*) due to OC use at young ages. According to a survey cited in TTG, 72% of the later cohorts (1951–1967) and about 31% of the earlier cohorts (1945–1950) stated that they had used OC before the age of 20. Assuming that OR* is the odds ratio for extended OC use (> 4 years) started before the age of 20 years, and OR is the odds ratio for extended OC use started after the age of 20 years, then the numbers in TTG can be expressed by the following equations:

$$0.31\ OR^* + 0.69\ OR = 0.8 \quad (1)$$
$$0.72\ OR^* + 0.28\ OR = 2.0 \quad (2)$$

Equations 1 and 2 represent the cohorts 1945–1950 and 1951–1967, respectively, and the numbers 0.8 and 2.0 are the weighted average (averaged over early and late starters) odds ratios shown in Table 3 in TTG. With 0.8 and 2.0 on the right-hand side, there is no solution when both OR and OR* are positive. If 0.8 in equation 1 was replaced with 1 we would get the solution OR = 0.24 and OR* = 2.68, i.e. a protection due to extended OC use when started after the age of 20 years and maybe a 10-fold (2.68/0.24) risk for early starters vs late starters. A ratio of that order is bound to have shown up in larger studies. These calculations are of course only approximations, but the message is that if there is a cohort effect as a result of different patterns in starting OC use, it is clear that the numbers on the right-hand side of equations 1 and 2 have to be much closer to 1 to get sensible results, i.e. there is little leeway for an eventual cohort difference on the basis of age difference when starting OC use.

In summary, these two Icelandic studies agree with the Collaborative Group on Hormonal Factors in Breast Cancer (1996) that the use of OC has very little impact on the risk for breast cancer. It could be that the odds ratio is greater than 1 for women who start using OC at a young age, but it seems to be very close to 1. The results of TT and TTG are compatible with results of the Collaborative Group (1996) that OC use does not have much impact, in either direction, on the cumulative incidence of breast cancer. A possible factor is that later cohorts might be diagnosed earlier in life. A confounding factor that is difficult to control for is that the women who start OC early might be subject to more medical evaluation, which is compatible with the Collaborative group (1996) who found that early OC users have a more benign form of cancer when diagnosed.

*H Tómasson,*
*University of Iceland,*
*Oddi v/ Sturlugötu,*
*IS-101 Reykjavik,*
*Iceland*

**REFERENCES**

Collaborative Group on Hormonal Factors in Breast Cancer (1996) Breast cancer and hormonal contraceptives: collaborative reanalysis of individual data on 53297 women with breast cancer and 100239 women without cancer from 54 epidemiological studies. *Lancet* **347**: 1713–1727

Tómasson H and Tómasson K (1996) Oral contraceptives and risk of breast cancer. A historical prospective case–control study. *Acta Obstet Gynecol Scand* **75**: 157–161

Tryggvadóttir L, Tulinius H and Gudmundsdóttir GB (1997) Oral contraceptives at young age and the risk of breast cancer: an Icelandic, population-based cohort study of the effect of birth year. *Br J Cancer* **75**: 139–143